# Machine Learning–Based Multidimensional Oximetry for Obstructive Sleep Apnea Screening: Development and External Validation

**DOI:** 10.2196/80384

**Published:** 2026-05-08

**Authors:** Xuanyu Qian, Haitong Luo, Rong Ding, Tianming Gao, Haoan Wang, Pengliang Wu, Ning Li

**Affiliations:** 1 Department of Respiratory and Critical Care Medicine Ruijin Hospital Shanghai Jiao Tong University School of Medicine Shanghai China; 2 Institute of Precision Optical Engineering School of Physics Science and Engineering Tongji University Shanghai China; 3 Department of Respiratory and Critical Care Medicine Taizhou Fourth People's Hospital Taizhou, Jiangsu Province China; 4 Department of Respiratory and Critical Care Medicine Pingliang Municipal Hospital of Traditional Chinese Medicine Pingliang, Gansu Province China; 5 Institute of Respiratory Diseases Shanghai Jiao Tong University School of Medicine Shanghai China

**Keywords:** obstructive sleep apnea, pulse oximetry, machine learning, multi-parameter oximetry, screening, CatBoost, categorical boosting

## Abstract

**Background:**

Obstructive sleep apnea (OSA) affects nearly one billion people globally and poses a substantial public health threat. Effective and accessible methods for OSA risk identification are urgently needed.

**Objective:**

This study aims to develop and externally validate a machine learning model derived from multi-parameter pulse oximetry (SpO_2_) for OSA screening, and to evaluate its performance, interpretability, and robustness across sex and age subgroups.

**Methods:**

Of 4156 screened participants, 2195 underwent polysomnography (internal cohort) and 446 received home sleep apnea testing (external cohort). Eight SpO_2_-derived parameters, including oxygen desaturation index (ODI), hypoxic burden (HB), and ST90 (percentage of sleep time with SpO_2_ < 90%), were used to construct models. Six machine learning algorithms were trained, with *F*_1_-score as the primary metric and area under the curve as the secondary metric. Model interpretability was assessed using Shapley additive explanations and intrinsic feature importance scores.

**Results:**

Nonlinear parameter-risk relationships were observed between oximetry indices and OSA probability. The 4-parameter ODI-HB-MinSpO_2_-ST90 model achieved optimal performance (*F*_1_-score = 0.9516, area under the curve = 0.9879), surpassing all single-parameter models. Shapley additive explanations analysis identified ODI, HB, and MinSpO_2_ as key predictors. The ODI-HB-MinSpO_2_-MeanSpO_2_ configuration demonstrated superior performance in female and younger subgroups, whereas the ODI-HB-MinSpO_2_-ST90 model remained optimal for male and older participants. Categorical boosting outperformed other algorithms across multiple metrics and remained robust in both subgroup and external validation analyses.

**Conclusions:**

The multi-parameter oximetry model based on the categorical boosting algorithm provides a simple and accurate tool for OSA screening. Sex- and age-stratified strategies can further enhance its clinical applicability.

## Introduction

Obstructive sleep apnea (OSA) affects nearly one billion individuals globally [1], and untreated OSA significantly increases the comorbidity burden and the risk of motor vehicle crashes [2]. Although polysomnography (PSG) remains the diagnostic gold standard, its high cost and operational complexity limit widespread accessibility [3,4]. Current screening tools, such as the STOP-BANG questionnaire or single physiological parameters, demonstrate limited diagnostic accuracy, with reported area under the receiver operating characteristic curve (AUC) values ranging from 0.55 to 0.83 [5,6]. Therefore, developing robust OSA screening tools using readily available physiological parameters remains imperative.

The pathophysiology of OSA is characterized by recurrent upper airway collapse, resulting in intermittent nocturnal hypoxia. Pulse oximetry-derived metrics, including the oxygen desaturation index (ODI), percentage of sleep time with SpO_2_ < 90% (ST90), and minimum oxygen saturation (MinSpO_2_), offer accessible alternatives to PSG [7], yet they reflect only a single dimension of nocturnal desaturation, thereby limiting their clinical utility [8]. ODI quantifies the frequency of desaturation events and correlates with PSG-derived apnea-hypopnea index (AHI), but does not capture hypoxic duration or desaturation depth [9]. ST90 reflects cumulative hypoxic burden (HB) but cannot distinguish between distinct hypoxic patterns, such as single prolonged versus multiple brief desaturations [9]. MinSpO_2_ identifies the instantaneous nadir but does not characterize cumulative hypoxic exposure [6]. The novel integrated HB metric, which combines desaturation depth, duration, and frequency, demonstrates superior predictive performance for OSA-related comorbidities compared with AHI and ODI [8], though direct comparisons with conventional metrics within the same datasets remain scarce [10]. Entropy and frequency-domain analyses of SpO_2_ complexity can capture dynamic nocturnal fluctuations overlooked by traditional metrics [11,12]. However, most existing studies evaluate parameters in isolation or focus on linear associations, leaving multidimensional feature integration and nonlinear relationships among parameters largely unexplored [4,13,14]. Thereby, multi-parameter models leveraging complementary oximetric indices may yield improved robustness for OSA screening [4,15,16].

Machine learning (ML) holds great potential for OSA diagnosis. Although deep learning models (eg, OxiNet) enable high-precision AHI estimation [17], their “black box” nature compromises clinical interpretability and raises clinical skepticism, thus limiting real-world utility [18,19]. Traditional ML algorithms show inconsistent performance across cohorts, with support vector machines (SVM) and random forests (RF) demonstrating variable performance [20,21]. Extreme gradient boosting (XGBoost) models for moderate-to-severe OSA exhibit limited accuracy (sensitivity 72.5%, specificity 62.8%) [22]. Least squares boosting for AHI estimation illustrates the benefits of ensemble approaches but does not resolve generalization issues between community and clinical cohorts [23]. Recent evidence suggests that categorical boosting (CatBoost) is superior for OSA classification, outperforming XGBoost, light gradient boosting machine (LightGBM), and RF in several studies [24,25], yet its application to oximetry-based OSA screening has not been evaluated.

Our study has three main aims: (1) to develop a parsimonious and robust OSA screening tool by evaluating multidimensional oximetric parameters using ML; (2) to validate model generalizability across community and clinical populations in an independent external cohort undergoing home sleep apnea test (HSAT); and (3) to assess performance heterogeneity across sex and age subgroups to inform personalized screening strategies.

## Methods

### Study Design and Population

**Figure 1:**
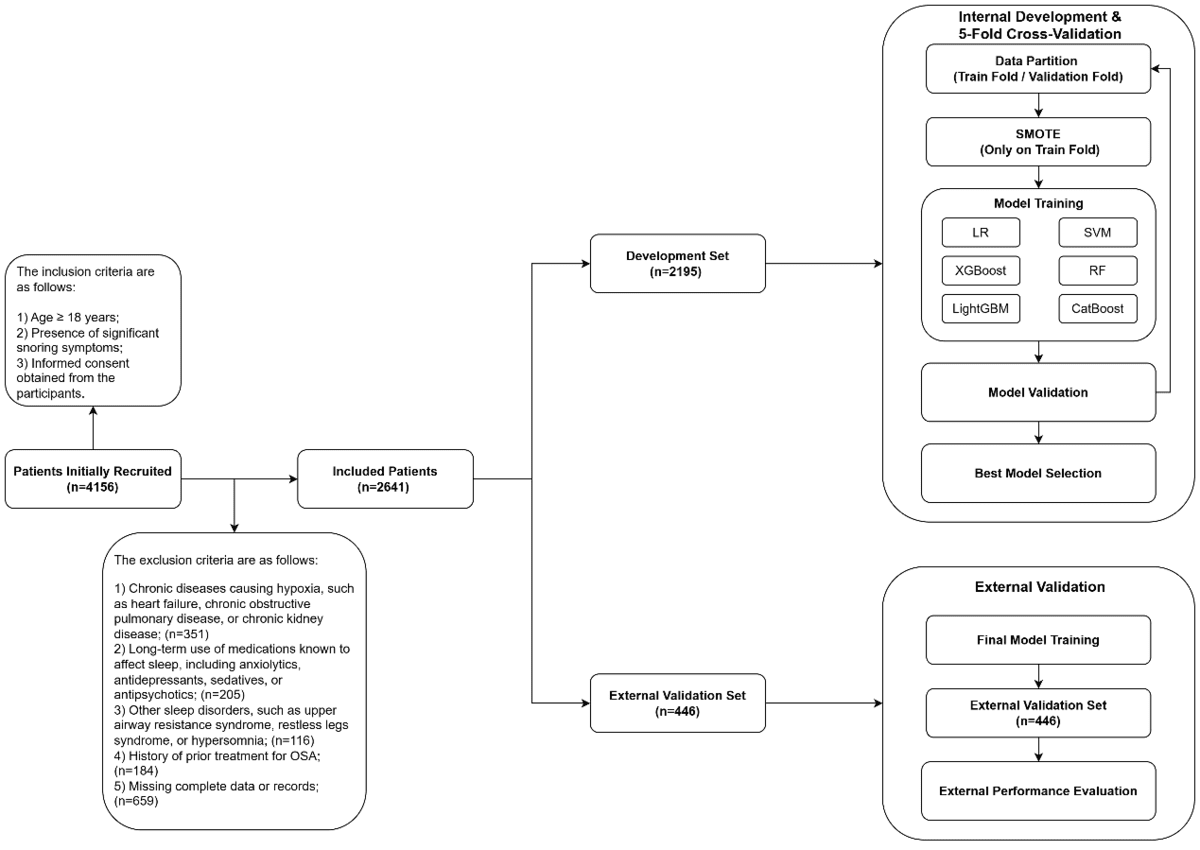
Flowchart of the overall study. CatBoost: categorical boosting; LightGBM: light gradient boosting machine; LR: logistic regression; OSA: obstructive sleep apnea; PSG: polysomnography; RF: random forest; SMOTE: synthetic minority over-sampling technique; SVM: support vector machine; XGBoost: extreme gradient boosting.

We consecutively enrolled adults with suspected OSA who underwent in-laboratory PSG at the Sleep Center of Ruijin Hospital, Shanghai Jiao Tong University School of Medicine, between June 2022 and July 2024. During the same period, we additionally recruited community-based participants who underwent HSAT. Inclusion criteria were age ≥18 years, prominent snoring, and provision of informed consent. Exclusion criteria included: (1) chronic diseases that may contribute to hypoxemia, such as heart failure, chronic obstructive pulmonary disease, chronic kidney disease; (2) chronic use of medications affecting sleep, including sedative-hypnotics, anxiolytics, antidepressants, and antipsychotics; (3) other concurrent sleep disorders, such as upper airway resistance syndrome, restless legs syndrome, or hypersomnia; (4) prior treatment for OSA; and (5) incomplete data. The participant flowchart is shown in Figure 1.

### Ethical Considerations

The study protocol complied with the Declaration of Helsinki and was approved by the Ruijin Hospital Ethics Committee (approval number: 2018-107). Due to its retrospective design, all data were fully deidentified, and the ethics committee waived the requirement for informed consent. All data were securely stored in accordance with institutional research data management standards, and no compensation was provided to participants.

### PSG and HSAT

Participants abstained from sedatives, alcohol, and caffeinated beverages for at least 24 hours prior to the study. In-laboratory PSG was performed using the Alice 6 system (Philips Respironics, Murrysville, PA, USA) with standard monitoring including electroencephalography, submental electromyography, bilateral electrooculography, electrocardiography, pulse oximetry, oronasal airflow via thermistor and pressure transducer, thoracoabdominal effort, snoring, and body position. HSAT used the Alice NightOne device (Philips Respironics, Murrysville, PA, USA) to record nasal airflow, respiratory effort, and fingertip SpO_2_. Recordings with more than 4 hours of analyzable data following manual review were considered valid. Two certified sleep specialists independently scored PSG and HSAT data according to the AASM scoring manual [3]: apnea was defined as ≥ 90% airflow reduction for ≥ 10 seconds, and hypopnea as ≥ 30% airflow reduction for ≥ 10 seconds accompanied by ≥ 4% SpO_2_ desaturation. AHI was calculated as the total number of apneas and hypopneas per hour of sleep, and OSA was defined as AHI ≥ 5 events/hour.

### Definition and Calculation of Pulse Oximetry Parameters

#### Summary of Signal Processing

During PSG and HSAT, SpO_2_ signals were collected at a sampling rate of 500 Hz and down-sampled to 1 Hz for computational efficiency. Eight parameters were extracted to quantify different aspects of nocturnal hypoxemia:

#### Mean SpO2 (MeanSpO2) and Minimum SpO2 (MinSpO2)

The average and the lowest SpO_2_ values during sleep:



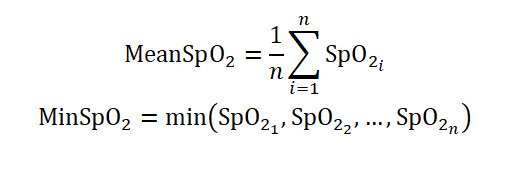



These reflect overall oxygenation status and the most severe desaturation [26].

#### Oxygen Desaturation Index (ODI)

Number of desaturation events (≥ 4% drop from baseline) per hour of sleep:



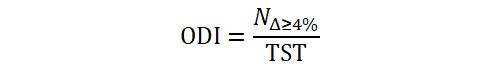



Where TST is the total sleep time (hours). ODI is a key indicator of the frequency of respiratory disturbances and serves as a surrogate for the AHI [27].

#### T90 and ST90

Total time and percentage of sleep spent with SpO_2_ below 90%:



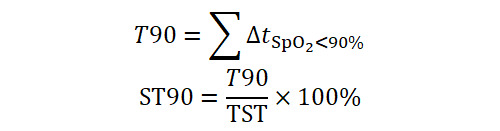



These quantify cumulative exposure to clinically significant hypoxemia [27].

#### Hypoxic Burden (HB)

**Figure 2:**
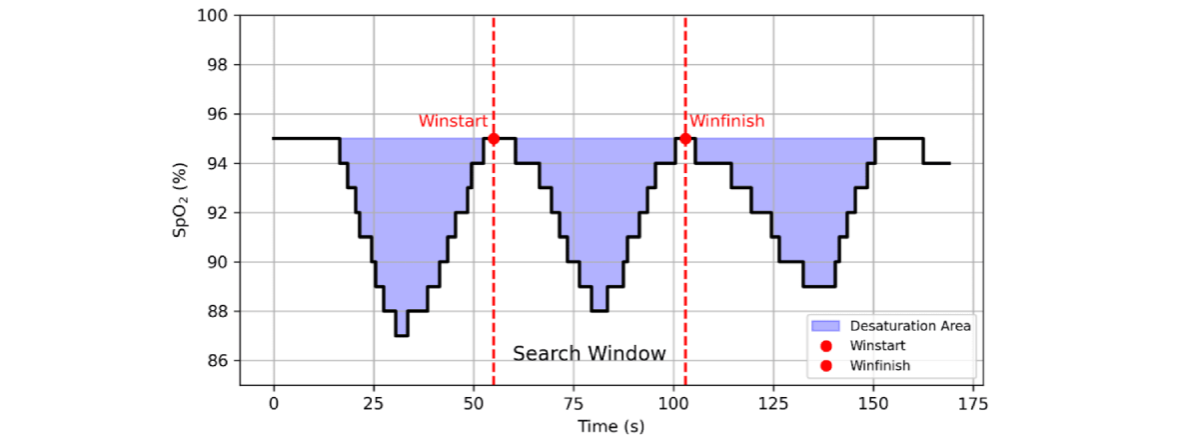
Hypoxia burden calculation using the rainwater collection algorithm applied to pulse oximetry signals.

The normalized total area under the SpO_2_ desaturation curve associated with respiratory events.



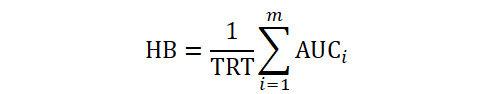



where AUC_i_ is the area of the *i*-th desaturation event identified by the “Trapping Rain Water” algorithm (Figure 2), and TRT is the total recording time. HB integrates frequency, depth, and duration of desaturations, representing total oxygen debt [28].

#### Attention Entropy (AttnEn)

AttnEn is a complexity measure of the SpO_2_ signal waveform variability [29].



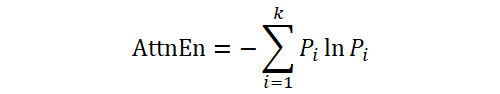



Where *P_i_* is the distribution of intervals between adjacent local extrema. Higher entropy reflects fragmented, unstable desaturation patterns typical of severe OSA.

#### Total Spectral Power (TotalPower)

Integrated Lomb–Scargle periodogram power within the ultradian band (0.014-0.035 Hz), corresponding to respiratory cycles of 30-70 seconds.



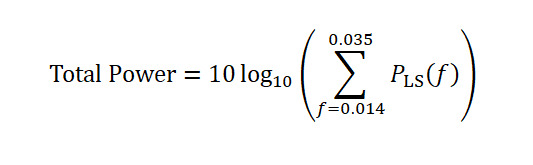



Elevated power in this band indicates the repetitive oscillatory desaturation dynamics characteristic of OSA [30,31].

The algorithm proceeds as follows: (1) Event Identification: The pulse oximetry (SpO_2_) signal is analyzed to detect all local minima (valleys), thereby identifying the nadir (lowest saturation) of each desaturation event. (2) Window Initialization: From each nadir, a bidirectional search is performed to delineate the event window (Win_start_ and Win_finish_). Boundaries are established at the nearest peaks that recover to ≥75% of the preceding peak-to-nadir amplitude. (3) Boundary Refinement: The search window is further adjusted based on the mean event duration to ensure temporal consistency. (4) Baseline Determination: The baseline for each event is defined as the maximum SpO_2_ value within the 100-second window preceding the event onset. (5) Area Integration: The under the curve (AUC for each event is computed by integrating the deficit between the baseline and the SpO_2_ signal within the defined window. (6) Hypoxia burden (HB) Calculation: All individual AUCs are summed to obtain the total desaturation area, which is then divided by the total recording time to derive the HB.

### Establishment and Validation of ML Models

#### Data Preprocessing

To mitigate bias from varying feature magnitudes, the data were first standardized via Z‑score normalization, transforming each feature to a mean of 0 and SD of 1 using:



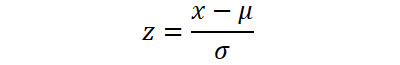



where μ and σ represent the mean and SD of the feature. This step ensures stable distance‑based computations and gradient optimization. Subsequently, class imbalance was addressed using the synthetic minority over‑sampling technique (SMOTE) [32,33]. SMOTE synthesizes minority‑class samples by interpolating between an instance *x_i_* and a randomly chosen neighbor *x̂_i_* from its *k*-nearest neighbors.



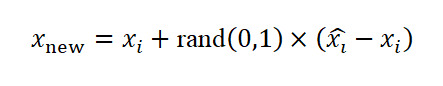



#### Algorithm Introduction

This study evaluates multiple ML models, grouped into three categories: (1) linear and kernel‑based models, (2) ensemble learning methods, and (3) gradient boosting decision trees, to balance interpretability with predictive performance. For linear and kernel-based models, logistic regression (LR) is a foundational model for clinical binary classification. It extends linear regression by applying the Sigmoid function to map linear outputs to a probability range between 0 and 1:



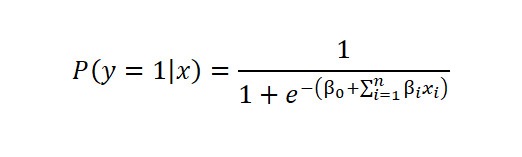



Where *P* is the predicted probability, *β*_0_ is the bias, *β_i_* are coefficients, and *x_i_* represent input features. Its transparency and low computational cost make it a standard benchmark in medical research [34,35].

SVM constructs an optimal separating hyperplane by maximizing the margin between classes. Its decision function is:

*w*·*x* + *b* = 0

Where *w* is the normal vector, *x* is the input feature, and *b* is the bias. The model is trained by minimizing 
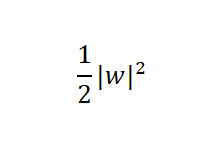
 subject to the constraint *y*_i_ (*w*·*x_i_* + *b*) ≥ 1, ensuring correct classification with a margin of at least one. SVMs excel in high-dimensional spaces and can capture nonlinear patterns through kernel functions, making them a widely adopted method [36].

For ensemble learning methods, RF is a bagging ensemble method that reduces overfitting by aggregating predictions from multiple decision trees. Each tree is trained on a bootstrap sample of the data and a random subset of features. The final prediction is obtained through majority voting:



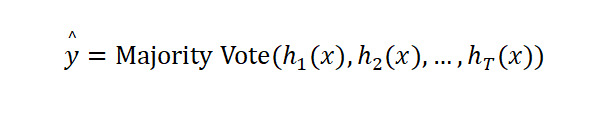



where *ŷ* is the final predicted result, *h*_t_(*x*) denotes the prediction of the *t*-th tree, and *T* is the total number of trees. By averaging across trees, RF improves stability and accuracy, making it effective for high-dimensional data and widely used in practice [37].

For gradient boosting decision trees, this kind of method iteratively combines weak learners, typically decision trees, to minimize a regularized objective function:



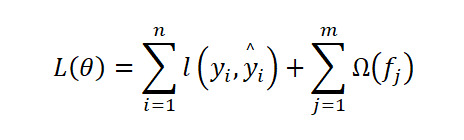



Where *l*(*y_i_*, *ŷ_i_*) represents the loss function, Ω(*f_j_*) controls model complexity; and *θ* denotes the parameters.

Three prominent variants, including XGBoost, LightGBM, and CatBoost, share this framework but differ in optimization and implementation: XGBoost uses second-order gradient approximation and explicit regularization, offering high precision and efficiency, especially with structured or sparse data [38,39]. LightGBM uses a leaf-wise growth strategy with gradient-based sampling and feature bundling, enabling faster training on large-scale datasets [40]. CatBoost is optimized for categorical features, using ordered target statistics and symmetric trees to prevent prediction shift and effectively handle high-dimensional categorical variables [41].

#### Modeling Process

The modeling pipeline followed a 2‑stage design: internal development with cross‑validation followed by independent external validation (Figure 1). In the internal phase, a cohort of 2195 subjects was preprocessed and evaluated using 5-fold cross-validation. To prevent data leakage, SMOTE was applied exclusively to the training folds, with validation sets retaining the original class distribution. Six ML algorithms were trained under fixed random seeds to ensure reproducibility, and hyperparameters are detailed in Table 1. Model selection was based on the average performance across validation folds. The best-performing model was subsequently retrained on the full internal dataset (n=2195) without SMOTE to preserve the original data distribution. The selected model’s generalization ability was then assessed on an independent external cohort (n=446). Performance on this external set reflects the model’s robustness for real-world OSA screening.

**Table 1:** Hyperparameters of the 6 machine learning models for obstructive sleep apnea screening.

Model	Hyperparameters
SVM^a^	‘C’: 1.0, ‘gamma’: ‘scale’, ‘kernel’: ‘rbf’
RF^b^	‘criterion’: ‘gini’, ‘max_features’: ‘sqrt’, ‘n_estimators’: 100
LR^c^	‘C’: 1.0, ‘penalty’: ‘l2’, ‘tol’ : 1e^–4^
XGBoost^d^	‘learning_rate’: 0.3, ‘reg_lambda’:1, ‘n_estimators’: 100, ‘booster’: ‘gbtree’
LightGBM^e^	‘learning_rate’: 0.1, ‘n_estimators’: 100, ‘boosting_type’: ‘gbdt’
CatBoost^f^	‘learning_rate’: 0.03, ‘n_estimators’: 100, ‘loss_function’: ‘Logloss’, ‘l2_leaf_reg’: 3

^a^SVM: support vector machine.

^b^RF: random forest.

^c^LR: logistic regression.

^d^XGBoost: extreme gradient boosting.

^e^LightGBM: light gradient boosting machine.

^f^CatBoost: categorical boosting.

#### Model Evaluation Metrics

Predictive performance was quantified using accuracy, sensitivity, specificity, *F*_1_-score, AUC, positive predictive value (PPV), and negative predictive value. The specific formulas are as follows:



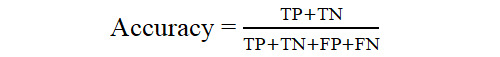





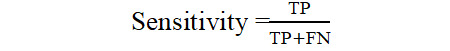





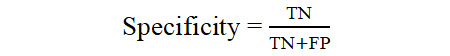





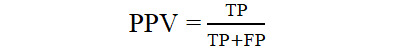





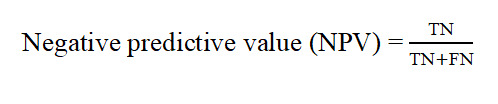





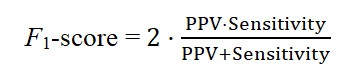



Where TP represents true positives, TN represents true negatives, FP represents false positives, and FN represents false negatives. Given the class imbalance in the clinical cohort, the *F*_1_-score was selected as the primary evaluation metric because it balances PPV and sensitivity (recall). In imbalanced clinical settings, AUC may overestimate performance by reflecting overall discriminability while masking poor sensitivity to the minority class. Unlike the threshold-independent AUC, the *F*_1_-score directly captures misclassification costs for minority samples, thereby ensuring robust diagnostic accuracy across classes. AUC is reported as a complementary measure of overall discriminative ability [42].

### Statistical Analysis

All analyses were conducted using Python (version 3.11; Python Software Foundation). Continuous variables are presented as median and IQR, and categorical variables as frequency and percentage. The Anderson–Darling test was used to assess normality. Group differences were evaluated with the Kruskal–Wallis H test, followed by Dunn’s post-hoc test (significance threshold *P*<.05). To examine linear and nonlinear associations between continuous predictors and the binary outcome, restricted cubic spline (RCS) regression was fitted within an LR framework. Likelihood-ratio tests compared RCS models against linear specifications, and spline curves were used to visualize dose-response relationships. To further interpret model predictions, Shapley additive explanations (SHAP) were used to quantify the contribution of each feature. Finally, stratified analyses by sex and age were conducted for these oximetry parameters.

## Results

### Characteristics of Study Participants

**Figure 3:**
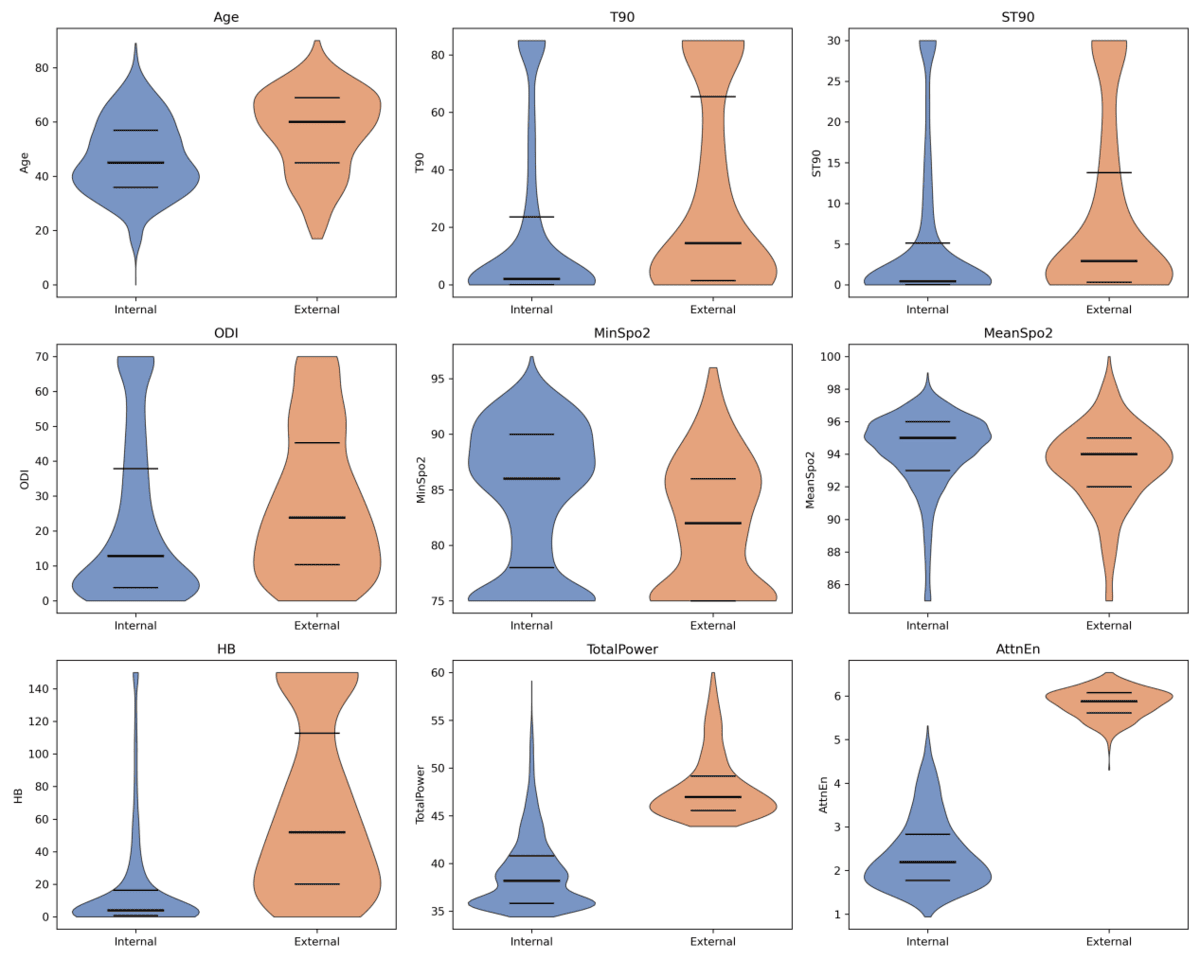
Comparison of baseline characteristics between the internal development and external validation cohorts. Violin plots comparing baseline characteristics between the internal development cohort (blue) and external validation cohort (orange). Each plot depicts the kernel density estimate, with bold horizontal lines representing medians and thin lines indicating IQRs. AttnEn: attention entropy; HB: hypoxia burden; MinSpO2: minimal SpO2; ODI: oxygen desaturation index; ST90: percentage of sleep time with SpO2 < 90%; T90: total sleep time spent with SpO2 < 90%; TotalPower: integrated power from power spectral density estimates in the 14-35 mHz frequency band.

Among 4156 screened participants, 2641 were included in the final analysis: 2195 undergoing PSG and comprised the internal development cohort, and 446 undergoing HSAT and formed the external validation cohort (Figure 1). The internal cohort consisted of 943 non-OSA and 1252 OSA participants. Compared with the non-OSA group, the OSA group was significantly older, a higher male proportion, experienced more frequent hypoxic episodes, and had longer hypoxic durations. The external cohort comprised 76 non-OSA and 370 OSA participants. These OSA patients displayed higher AHI, ODI, ST90, T90, and HB values alongside lower oxygen saturation, yet they were younger than non-OSA participants, with no significant between-group difference in sex distribution. Demographic and clinical characteristics are summarized in Table 2. Violin plots (Figure 3) revealed a higher median age (60.0 vs 45.0 years) and more severe nocturnal hypoxemia in the external validation cohort, underscoring distinct disease severity and physiological profiles between the 2 cohorts. These differences provide a robust foundation for validating the generalizability of the multi-parameter oximetry model across diverse clinical scenarios.

**Table 2:** Baseline characteristics of non-obstructive sleep apnea and obstructive sleep apnea patients in the internal development and external validation cohorts.

Characteristics	Internal development cohort					External cohort			
	All (n=2195)	Non-OSA^a^ (n=953)	OSA (n=1242)	*P* value	All (n=446)		Non-OSA (n=76)	OSA (n=370)	*P* value
Age (years), median (IQR)	45.00 (36.00-57.00)	45.00 (35.00-57.00)	46.00 (37.00-57.00)	.001	60.0 (45.00-69.00)		63.00 (49.75-71.25)	58.00 (44.00-68.00)	<.001
Male, n (%)	1651 (75.22)	684 (71.77)	967 (77.86)	<.001	351 (78.70)		55 (72.37)	296 (80.00)	.14
AHI^b^ (events/h), median (IQR)	8.20 (2.10-34.10)	1.80 (0.90-3.00)	29.15 (13.83-54.08)	<.001	19.40 (9.03-37.48)		2.25 (0.90-3.58)	24.80 (13.65-42.45)	<.001
ODI^c^ (events/h), median (IQR)	12.80 (3.80-37.90)	3.30 (1.70-5.40)	33.45 (16.70-58.55)	<.001	23.85 (10.40-45.28)		3.40 (1.18-5.13)	29.00 (17.35-49.53)	<.001
MinSpO_2_^d^ (%), median (IQR)	86.00 (78.00-90.00)	90.00 (88.00-92.00)	79.00 (71.00-85.00)	<.001	82.00 (75.00-86.00)		89.00 (88.00-91.00)	80.00 (72.00-85.00)	<.001
MeanSpO_2_ (%), median (IQR)	95.00 (93.00-96.00)	96.00 (95.00-96.00)	94.00 (92.00-95.00)	<.001	94.00 (92.00-95.00)		95.00 (94.00-97.00)	93.00 (92.00-95.00)	<.001
ST90^e^ (%), median (IQR)	0.44 (0.02-5.12)	0.02 (0-0.11)	3.38 (0.72-14.42)	<.001	2.91 (0.32-13.80)		0.0 (0-0.20)	4.51 (1.12-16.48)	<.001
T90^f^ (minute), median (IQR)	2.00 (0.10-23.70)	0.10 (0.00-0.50)	16.20 (3.5-67.10)	<.001	14.50 (1.53-65.52)		0.00 (0.00-0.10)	22.20 (5.55-78.9)	<.001
HB^g^ (%·min/h), median (IQR)	3.90 (0.90-16.40)	0.70 (0.20-1.70)	13.20 (5.50-36.80)	<.001	51.83 (20.22-112.84)		6.29 (2.32-9.70)	65.97 (35.07-138.08)	<.001
AttnEn^h^, median (IQR)	2.19 (1.78-2.84)	1.74 (1.54-1.97)	2.70 (2.25-3.37)	<.001	5.87 (5.62-6.10)		6.10 (5.99-6.25)	5.82 (5.57-6.01)	<.001
TotalPower^i^ (dB), median (IQR)	38.17 (35.83-40.81)	37.61 (35.62-40.64)	38.52 (36.10-41.10)	<.001	46.95 (45.56-49.16)		45.06 (44.81-45.40)	47.33 (46.11-50.28)	<.001

^a^OSA: obstructive sleep apnea.

^b^AHI: apnea-hypopnea index.

^c^ODI: oxygen desaturation index.

^d^MinSpO_2_: minimal SpO_2_.

^e^ST90: percentage of sleep time with SpO_2_ < 90%.

^f^T90: total sleep time spent with SpO_2_ < 90%.

^g^HB: hypoxia burden.

^h^AttnEn: attention entropy.

^i^TotalPower: integrated power from power spectral density estimates in the 14-35 mHz frequency band.

### Performance of Single-Parameter Oximetry Models

**Figure 4:**
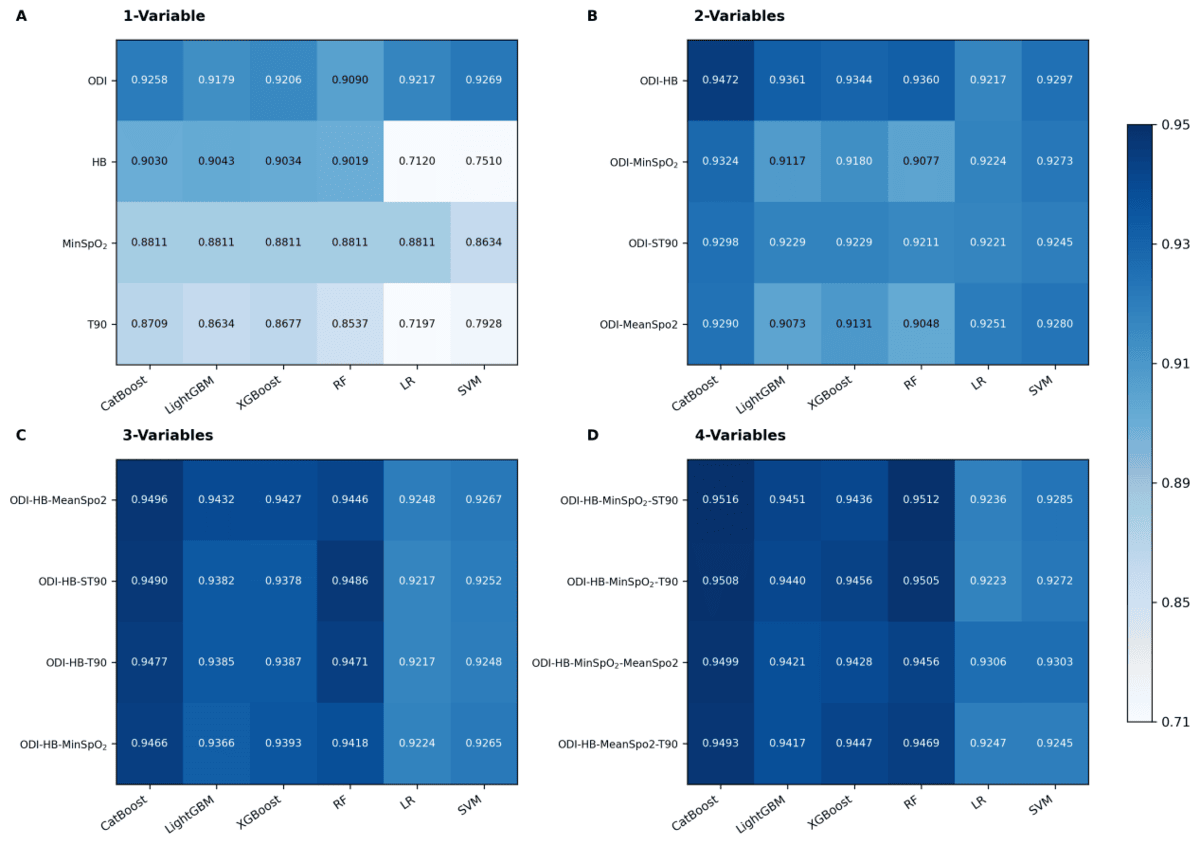
Heatmap of F1-scores for multi-parameter oximetry models across 6 machine learning algorithms. (A) single-parameter; (B-D) combinations of 2, 3, and 4 parameters, respectively. The top 4 F1-scores are shown for each model configuration, with darker colors representing higher classification performance. CatBoost: categorical boosting; HB: hypoxia burden; LightGBM: light gradient boosting machine; LR: logistic regression; MinSpO2: minimal SpO2; ODI: oxygen desaturation index; OSA: obstructive sleep apnea; RF: random forest; ST90: percentage of sleep time with SpO2 <90%; SVM: support vector machine; T90: total sleep time spent with SpO2 < 90%; XGBoost: extreme gradient boosting.

We evaluated the predictive performance of 8 OSA-related oximetry parameters using 6 ML algorithms. Given the class imbalance between OSA and non-OSA groups in the internal cohort, the *F*_1_-score was selected as the primary metric to balance precision and recall, with AUC used to assess overall discriminative performance [43]. Substantial heterogeneity was observed in model performance, with *F*_1_-scores ranging from 0.5332 to 0.9269 and AUC values from 0.5660 to 0.9808. Notably, ODI and HB exhibited the strongest discriminative ability. Figure 4A summarizes the top 4 single-parameter oximetry models ranked by *F*_1_-score. The SVM model achieved optimal performance for ODI (*F*_1_-score = 0.9269, AUC = 0.9712), and the LightGBM model performed best for HB (*F*_1_-score = 0.9043, AUC = 0.9590). By contrast, MeanSpO_2_ and TotalPower showed comparatively weaker discriminative capacity, with *F*_1_-score of 0.7073 (LR model) and 0.6713 (CatBoost model), respectively.

**Figure 5:**
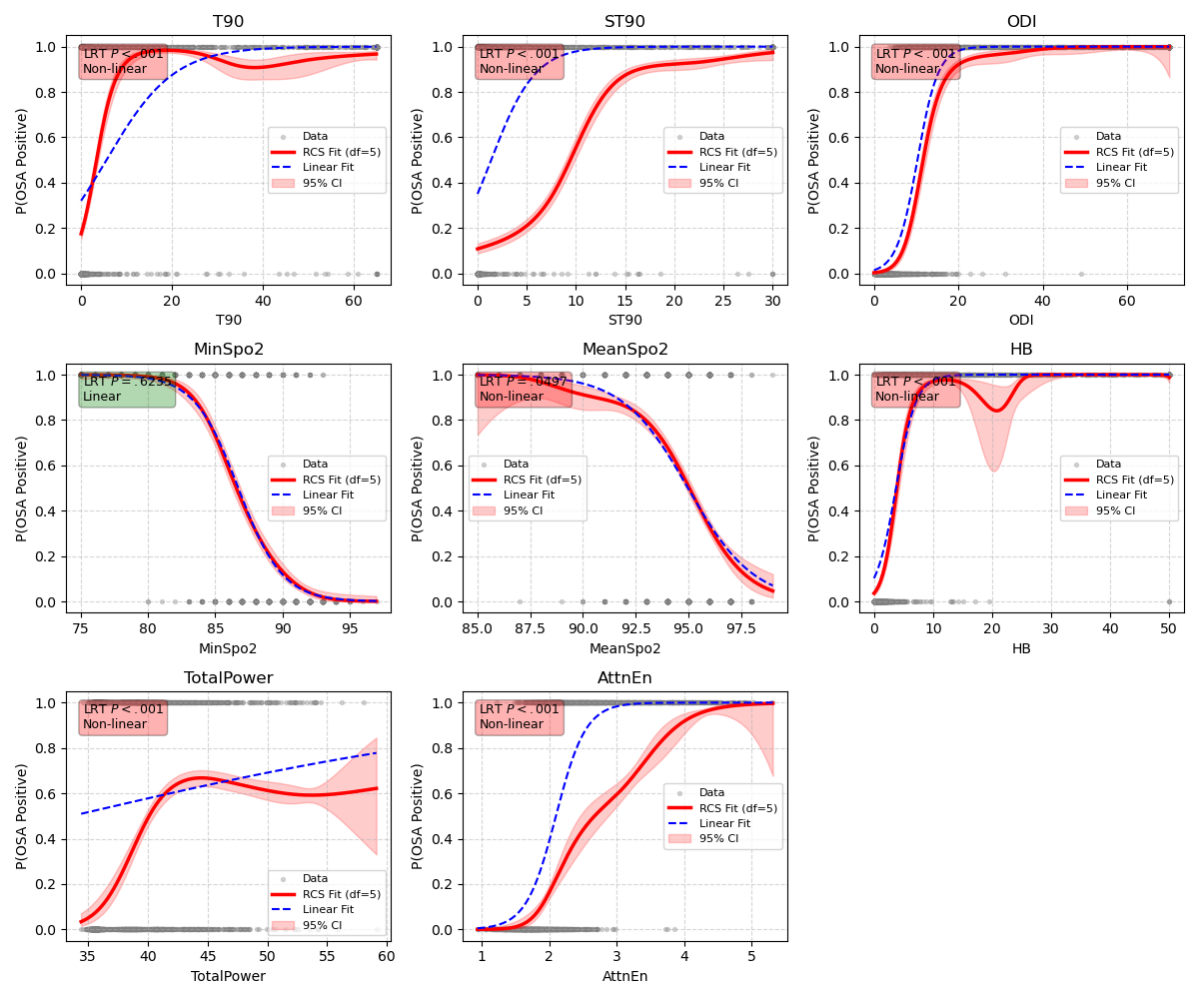
RCS curves showing associations between oximetry parameters and OSA risk. The analysis was performed on the internal development cohort (n=2195). The solid red lines indicate RCS fits with 5 degrees of freedom, and the red shaded areas indicate the 95% CIs. The blue dashed lines represent the linear fit for comparison. The y-axis represents the predicted probability of OSA. The *P* values were derived from Likelihood Ratio Tests to evaluate nonlinearity (nonlinearity: *P*<.05, red boxes; linear: P≥.05, green boxes). The gray dots (top and bottom) represent individual data distributions for OSA-positive and OSA-negative participants, respectively. AttnEn: attention entropy; HB: hypoxia burden; MinSpO2: minimal SpO2; ODI: oxygen desaturation index; OSA: obstructive sleep apnea; RCS: restricted cubic spline; ST90: percentage of sleep time with SpO2 <90%; T90: total sleep time spent with SpO2 < 90%; TotalPower: integrated power from power spectral density estimates in the 14-35 mHz frequency band.

Beyond the linear association of MinSpO_2_, all other oximetry parameters exhibited nonlinear relationships with OSA risk (*P*<.001), accounting for the heterogeneous predictive performance across indicators. Strong predictors, including ODI, HB, T90, and ST90, exhibited steep dose-response curves with pronounced threshold effects (Figure 5). For instance, ODI showed a rapid risk escalation at lower values followed by a plateau, thereby providing distinct decision boundaries that enhanced the model’s discriminative ability and optimized *F*_1_-scores. Conversely, weaker predictors exhibited contrasting profiles: MeanSpO_2_ showed shallow gradients within the clinically critical 88%-92% range, resulting in classification ambiguity, whereas TotalPower displayed marked variability with widened 95% CIs at higher values, indicating substantial noise that limited predictive utility (Figure 5). Given the complex nonlinear patterns of most key predictors, traditional linear regression models fail to capture these critical features.

### Predictive Performance of Multi-Parameter Oximetry Models

**Figure 6:**
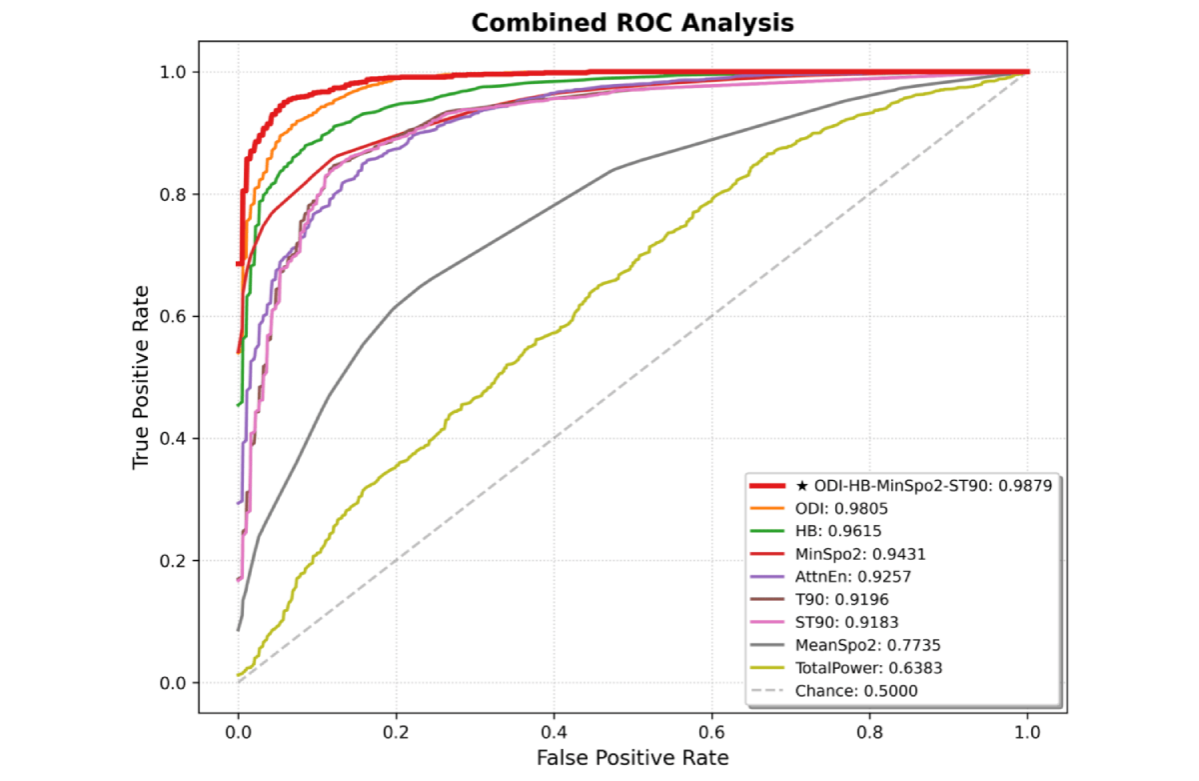
ROC curves of the optimal 4-parameter oximetry model versus single-parameter oximetry models for OSA screening. AUC was used to quantify model discrimination, with values closer to 1 indicating better predictive ability. AttnEn: attention entropy; AUC: area under the receiver operating characteristic curve; HB: hypoxia burden; MinSpO2: minimal SpO2; ODI: oxygen desaturation index; OSA: obstructive sleep apnea; ROC: receiver operating characteristic; ST90: percentage of sleep time with SpO2 < 90%; T90: total sleep time spent with SpO2 < 90%; TotalPower: integrated power from power spectral density estimates in the 14-35 mHz frequency band.

We constructed and evaluated multi-parameter oximetry models, including 28 dual-, 56 triple-, and 70 quadruple-parameter combinations, with top-performing models illustrated in Figure 4B-D. Among the dual-parameter models, the CatBoost-trained ODI-HB model achieved optimal performance (*F*_1_-score = 0.9472, AUC = 0.9865; Table 3, Figure 4B). The ODI-HB-MinSpO_2_ model performed best in the triple-parameter category (*F*_1_-score = 0.9496, AUC = 0.9869; Table 3, Figure 4C), whereas the quadruple-parameter ODI-HB-MinSpO_2_-ST90 model attained the highest overall discriminative ability (*F*_1_-score = 0.9516, AUC = 0.9879), significantly outperforming single-parameter oximetry models (Table 3, Figure 6). CatBoost demonstrated consistent superiority across all evaluation metrics (Table 3). Notably, adding 5 or more oximetry parameters yielded only marginal gains, underscoring the importance of selecting informative and complementary features rather than an indiscriminate increase in input dimensionality.

**Table 3:** Comparison of machine learning algorithms for obstructive sleep apnea screening using multi-parameter oximetry.

Feature sets and machine learning model			AUC^a^		*F*_1_-score		Accuracy		Sensitivity	Specificity		PPV^b^		NPV^c^
**ODI-HB^d,e^**														
	CatBoost^f^	0.9865		0.9472		0.9408		0.9412		0.9402	0.9537		0.9253	
	LightGBM^g^	0.9280		0.9361		0.9280		0.9332		0.9213	0.9396		0.9143	
	XGBoost^h^	0.9812		0.9344		0.9262		0.9300		0.9213	0.9392		0.9104	
	RF^i^	0.9794		0.9360		0.9280		0.9316		0.9234	0.9409		0.9129	
	LR^j^	0.9809		0.9217		0.9134		0.8881		0.9496	0.9586		0.8655	
	SVM^k^	0.9774		0.9297		0.9226		0.9066		0.9434	0.9545		0.8863	
**ODI-HB-MeanSpO_2_^l^**														
	CatBoost	0.9869		0.9496		0.9435		0.9420		0.9454	0.9575		0.9265	
	LightGBM	0.9848		0.9432		0.9367		0.9332		0.9413	0.9540		0.9165	
	XGBoost	0.9831		0.9427		0.9358		0.9372		0.9339	0.9487		0.9205	
	RF	0.9803		0.9446		0.9380		0.9348		0.9423	0.9550		0.9179	
	LR	0.9816		0.9248		0.9180		0.8921		0.9517	0.9604		0.8720	
	SVM	0.9796		0.9267		0.9194		0.9018		0.9423	0.9536		0.8813	
**ODI-HB-MinSpO_2_-ST90^m^**														
	CatBoost	0.9879		0.9516		0.9458		0.9444		0.9475	0.9592		0.9296	
	LightGBM	0.9862		0.9451		0.9385		0.9388		0.9381	0.9520		0.9227	
	XGBoost	0.9842		0.9436		0.9367		0.9380		0.9349	0.9496		0.9212	
	RF	0.9856		0.9512		0.9453		0.9444		0.9465	0.9585		0.9299	
	LR	0.9811		0.9236		0.9162		0.8970		0.9412	0.9525		0.8760	
	SVM	0.9815		0.9285		0.9212		0.9050		0.9423	0.9537		0.8844	

^a^AUC: area under the receiver operating characteristic curve.

^b^PPV: positive predictive value.

^c^NPV: negative predictive value.

^d^ODI: oxygen desaturation index.

^e^HB: hypoxia burden.

^f^CatBoost: categorical boosting.

^g^LightGBM: light gradient boosting machine.

^h^XGBoost: extreme gradient boosting.

^i^RF: random forest.

^j^LR: logistic regression.

^k^SVM: support vector machine.

^l^MinSpO_2_: minimal SpO_2_.

^m^ST90: percentage of sleep time with SpO_2_ < 90%.

### Stratified Analysis by Sex and Age

**Figure 7:**
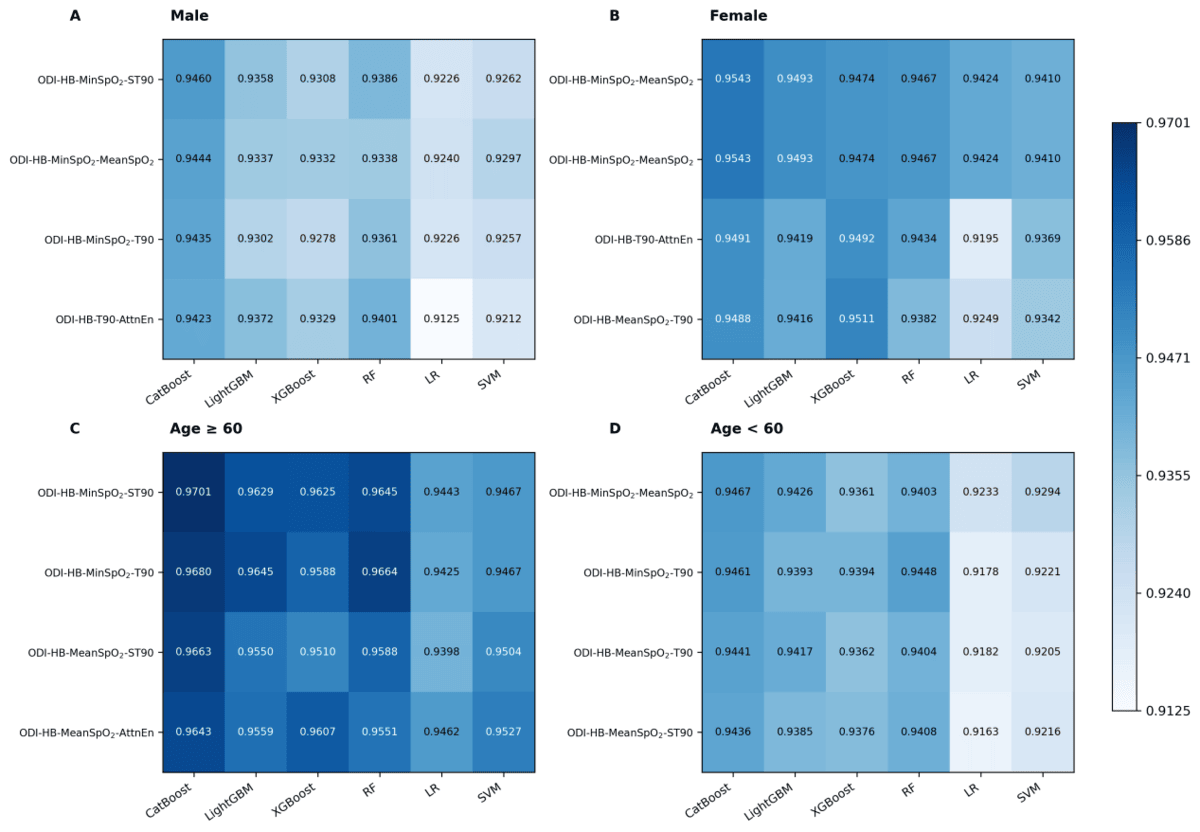
Heatmap of F1-scores for 4-parameter oximetry models across age and sex subgroups. (A) Male subgroup; (B) female subgroup; (C) older subgroup (≥ 60 years); (D) younger subgroup (< 60 years). The top 4 F1-scores are displayed for each subgroup, with darker colors indicating superior classification performance. AttnEn: attention entropy; CatBoost: categorical boosting; HB: hypoxia burden; LightGBM: light gradient boosting machine; LR: logistic regression; MinSpO2: minimal SpO2; ODI: oxygen desaturation index; OSA: obstructive sleep apnea; RF: random forest; ST90: percentage of sleep time with SpO2 <90%; SVM: support vector machine; T90: total sleep time spent with SpO2 < 90%; XGBoost: extreme gradient boosting.

Subgroup analyses revealed significant performance variations across demographics. In the male subgroup, the optimal model (ODI-HB-MinSpO_2_-ST90) achieved an *F*_1_-score of 0.9460 and an AUC of 0.9853, with CatBoost outperforming other algorithms (Table 4, Figure 7A). In the female subgroup, the best-performing combination was ODI-HB-MinSpO_2_-MeanSpO_2_ (*F*_1_-score = 0.9543, AUC = 0.9919; Table 4, Figure 7B), suggesting sex-specific differences in OSA-related hypoxic patterns. In the age-stratified analysis, the older subgroup demonstrated superior overall performance (*F*_1_-score = 0.9398-0.9701, AUC = 0.9913-0.9933) with ODI-HB-MinSpO_2_-ST90 as the optimal model, whereas the younger subgroup exhibited stable but slightly lower performance (*F*_1_-score = 0.9163-0.9467, AUC = 0.9774-0.9863), favoring ODI-HB-MinSpO_2_-MeanSpO_2_ (Figure 7C-D). Across all subgroups, CatBoost maintained consistently superior classification performance (Table 4).

**Table 4:** Performance of optimal predictive models for obstructive sleep apnea screening across sex and age subgroups in the internal development cohort.

Feature Sets	Subgroup^a^	AUC^b^	*F*_1_-score	Accuracy	Sensitivity	Specificity	PPV^c^	NPV^d^
ODI-HB-MinSpO_2_-ST90^e,f,g,h^	Male	0.9853	0.9460	0.9376	0.9338	0.9438	0.9587	0.9102
ODI-HB-MinSpO_2_-MeanSpO_2_	Female	0.9919	0.9543	0.9541	0.9527	0.9554	0.9572	0.9532
ODI-HB-MinSpO_2_-ST90	Older (≥ 60 years)	0.9942	0.9701	0.9657	0.9664	0.9647	0.9741	0.9552
ODI-HB-MinSpO_2_-MeanSpO_2_	Younger (< 60 years)	0.9863	0.9467	0.9404	0.9384	0.9429	0.9552	0.9224

^a^All subgroup models used CatBoost as the optimal classifier.

^b^AUC: area under the receiver operating characteristic curve.

^c^PPV: positive predictive value.

^d^NPV: negative predictive value.

^e^ODI: oxygen desaturation index.

^f^HB: hypoxia burden.

^g^MinSpO_2_: minimal SpO_2_.

^h^ST90: percentage of sleep time with SpO_2_ < 90%.

### Model Interpretability

**Figure 8:**
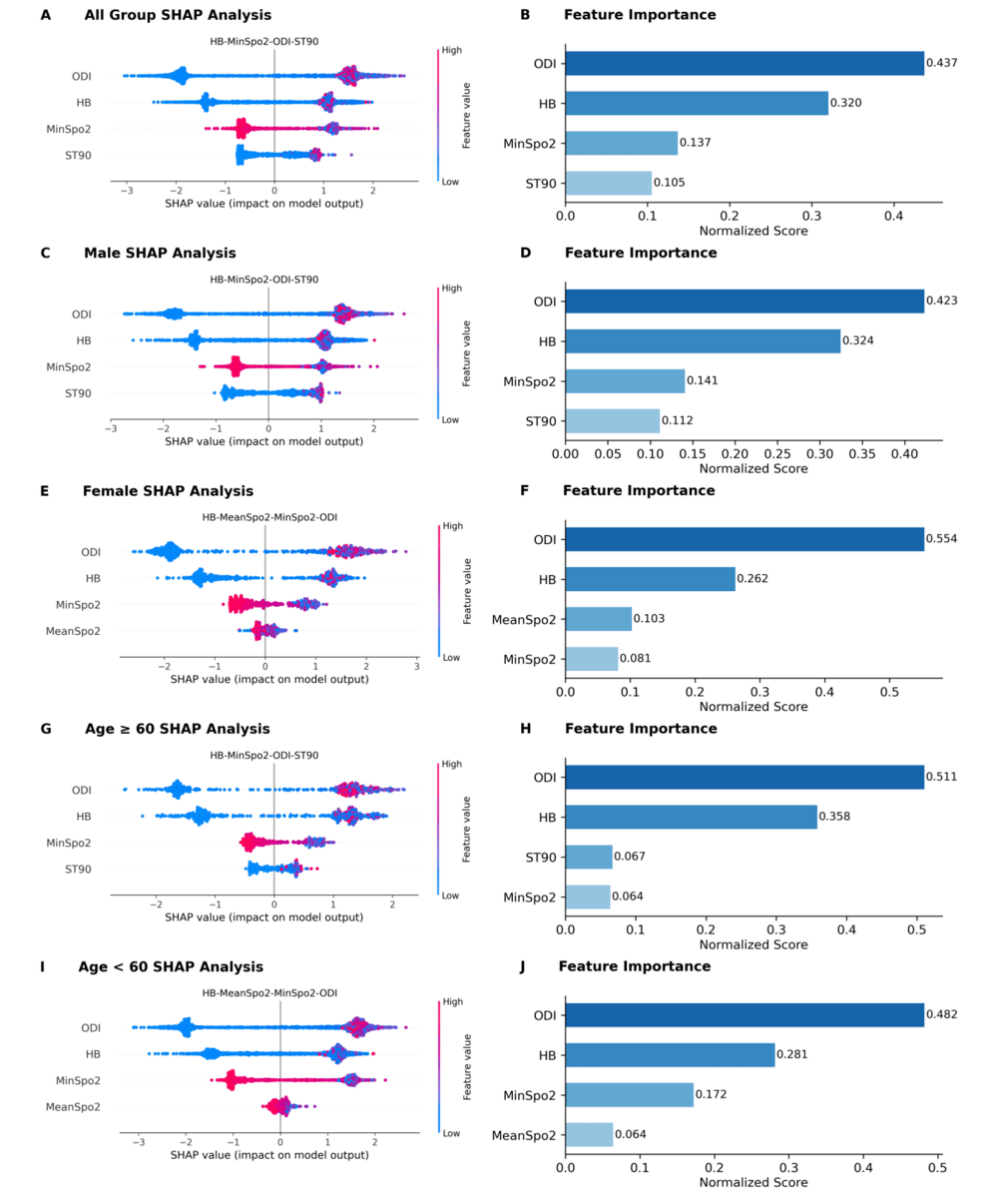
Interpretability analysis of oximetry parameters across sex and age subgroups for OSA screening. (A, C, E, G, I) SHAP summary plots illustrating feature contributions; dot color denotes feature magnitude (red: high, blue: low) and horizontal position indicates the SHAP value. (B, D, F, H, J) Normalized feature importance scores. Results are presented for all participants (A,B), male subgroup (C,D), female subgroup (E,F), older subgroup (≥ 60 years) (G,H), and younger subgroup (< 60 years) (I,J). HB: hypoxia burden; MinSpO2: minimal SpO2; ODI: oxygen desaturation index; OSA: obstructive sleep apnea; SHAP: Shapley additive explanations; ST90: percentage of sleep time with SpO2 < 90%.

To elucidate the predictive mechanisms of the optimal 4-parameter model, we integrated SHAP analysis with normalized feature importance scores. SHAP values quantified each feature’s marginal contribution and revealed nonlinear relationships between oximetry parameters and OSA risk (Figure 8A), while normalized scores reflected relative contribution weights (Figure 8B). In the internal cohort, ODI, HB, and MinSpO_2_ emerged as the top 3 predictors, with importance scores of 0.437, 0.320, and 0.137, respectively (Figure 8B). Subgroup analyses revealed heterogeneous contribution patterns across sex and age strata (Figure 8C-J). Notably, male and older subgroups showed consistent dominance of ODI, HB, MinSpO_2_, and ST90 (Figure 8C, D, G, H), with ODI exhibiting the highest contribution in the older subgroup (importance score: 0.511). Conversely, younger and female subgroups were characterized by ODI-HB-MinSpO_2_-MeanSpO_2_, where MeanSpO_2_ replaced ST90 as a stronger predictor (Figure 8E,F,I,J). Particularly in females, MeanSpO_2_ surpassed MinSpO_2_ in contribution strength (Figure 8F).

### External Validation

**Figure 9:**
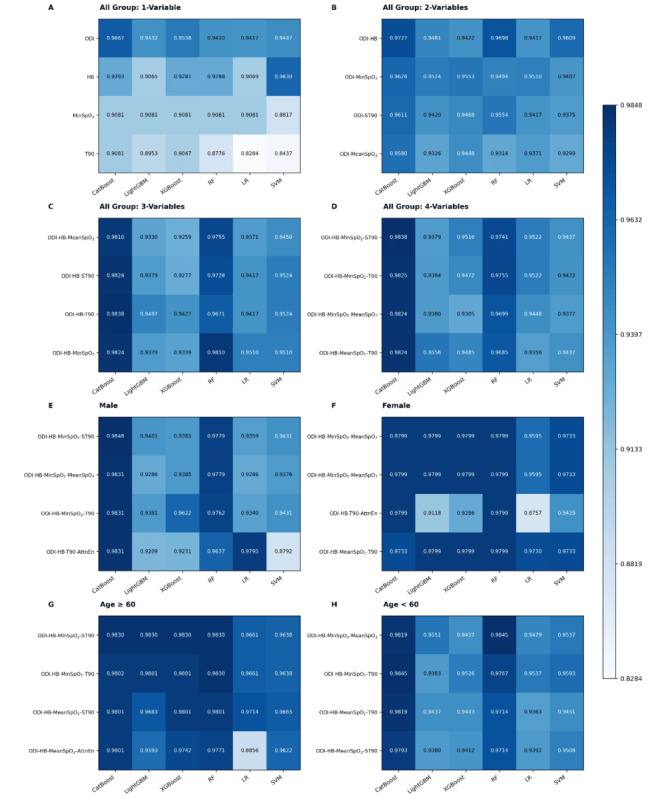
Heatmap of F1-scores in external validation across parameter combinations and demographic subgroups. (A-D) Performance in all participants by parameter complexity: (A) single-parameter, (B) 2-parameter, (C) 3-parameter, and (D) 4-parameter models. (E-H) Performance of 4-parameter oximetry models across subgroups: (E) male, (F) female, (G) older (≥ 60 years), and (H) younger (< 60 years). The heatmap displays the top 4 F1-scores for each parameter combination across 6 machine learning algorithms. Darker colors indicate higher F1-scores, reflecting superior classification performance. AttnEn: attention entropy; CatBoost: categorical boosting; HB: hypoxia burden; LightGBM: light gradient boosting machine; LR: logistic regression; MinSpO2: minimal SpO2; ODI: oxygen desaturation index; RF: random forest; ST90: percentage of sleep time with SpO2 < 90%; SVM: support vector machine; T90: total sleep time spent with SpO2 < 90%; XGBoost: extreme gradient boosting.

To assess generalizability, we tested model performance on an independent external cohort. The CatBoost algorithm demonstrated robust generalizability, achieving an *F*_1_-score of 0.9667 with single-parameter configurations and maintaining high performance as oximetry parameter complexity increased (Table 5). Specifically, the optimal 4-parameter oximetry model (ODI-HB-MinSpO_2_-ST90) achieved an *F*_1_-score of 0.9838 and an AUC of 0.9881 (Table 5, Figure 9D), suggesting that the model captures shared OSA pathophysiological features rather than overfitting the internal cohort. Subgroup analysis further confirmed the robustness of sex- and age-stratified models in the external cohort (Table 6). Sex-optimized models achieved *F*_1_-scores of 0.9848 (male subgroup: ODI-HB-MinSpO_2_-ST90) and 0.9799 (female subgroup: ODI-HB-MinSpO_2_-MeanSpO_2_), with AUCs exceeding 0.98 (Figure 9E, F). Age-stratified models similarly achieved *F*_1_-scores exceeding 0.98 across subgroups (Figure 9G, H). These results validate the excellent generalizability of the CatBoost-based oximetry model for diverse OSA screening applications (Table 6).

**Table 5:** Performance of multi-parameter oximetry models in external validation. All subgroup models used categorical boosting (CatBoost) as the optimal classifier.

Feature sets	AUC^a^	*F*_1_-score	Accuracy	Sensitivity	Specificity	PPV^b^	NPV^c^
ODI^d^	0.9877	0.9667	0.9462	0.9405	0.9737	0.9943	0.7708
ODI-HB^e^	0.9861	0.9727	0.9552	0.9622	0.9211	0.9834	0.8333
ODI-HB-MeanSpO_2_^f^	0.9863	0.9810	0.9686	0.9784	0.9211	0.9837	0.8974
ODI-HB-MinSpO_2_-ST90^g^	0.9881	0.9838	0.9731	0.9838	0.9211	0.9838	0.9211

^a^AUC: area under the receiver operating characteristic curve.

^b^PPV: positive predictive value.

^c^NPV: negative predictive value.

^d^ODI: oxygen desaturation index.

^e^HB: hypoxia burden.

^f^MinSpO_2_: minimal SpO_2_.

^g^ST90: percentage of sleep time with SpO_2_ < 90%.

**Table 6:** Performance of optimal predictive models in external validation across sex and age subgroups. All subgroup models used categorical boosting (CatBoost) as the optimal classifier.

Feature sets	Subgroup	AUC^a^	*F*_1_-score	Accuracy	Sensitivity	Specificity	PPV^b^	NPV^c^
ODI-HB-MinSpO_2_-ST90^d,e,f,g^	Male	0.9854	0.9848	0.9744	0.9831	0.9273	0.9864	0.9107
ODI-HB-MinSpO_2_-MeanSpO_2_	Female	0.9916	0.9799	0.9684	0.9865	0.9048	0.9733	0.9500
ODI-HB-MinSpO_2_-ST90	Older (≥ 60 years)	0.9855	0.9830	0.9733	0.9719	0.9787	0.9943	0.9020
ODI-HB-MinSpO_2_-MeanSpO_2_	Younger (< 60 years)	0.9914	0.9819	0.9683	0.9896	0.8276	0.9774	0.9231

^a^AUC: area under the receiver operating characteristic curve.

^b^PPV: positive predictive value.

^c^NPV: negative predictive value.

^d^ODI: oxygen desaturation index.

^e^HB: hypoxia burden.

^f^MinSpO_2_: minimal SpO_2_.

^g^ST90: percentage of sleep time with SpO_2_ < 90%.

## Discussion

### Principal Findings and Model Development

This study presents the first comprehensive evaluation of multidimensional oximetric parameters for OSA screening. Using 6 ML algorithms, we developed and rigorously validated an integrated multi-parameter model that overcomes the inherent limitations of conventional single- or dual-parameter approaches [44,45]. Through a novel algorithm-parameter matching framework, we established a CatBoost model combining ODI, HB, MinSpO_2_, and ST90. This model showed robust performance in external validation, providing a streamlined and high-precision tool for OSA screening. Furthermore, our findings elucidate the heterogeneous contributions of oximetric parameters across sex- and age-specific subgroups, addressing a critical gap in population-specific research [46] and providing the foundation for personalized risk stratification.

### Model Generalizability and Oximetric Parameter Performance

We used a large internal development set derived from PSG and an independent external validation set derived from HSAT, which included older patients with severe nocturnal hypoxemia, representing distinct clinical phenotypes. This integration of community and clinical data improves model generalizability and may streamline OSA diagnosis [4,23]. Consistent with previous reports, HSAT showed high diagnostic accuracy and strong correlation with PSG in older patients with severe OSA [47]. As expected, the OSA group was predominantly male and exhibited worse oximetry profiles [1,22,23]. Single-parameter oximetry models demonstrated marked variability in predictive performance, with ODI and HB emerging as the strongest predictors. ODI quantifies hourly desaturation frequency and correlates strongly with AHI, acting as an independent predictor of OSA severity regardless of sleep stage or body position [48,49]. Although ODI >20 events/h showed high sensitivity (96.6%) for severe OSA, its AUC for mild disease was only 0.62, suggesting limited standalone utility [50,51]. In contrast, HB integrates desaturation depth and duration, better capturing the cumulative physiological burden of intermittent hypoxemia [44,46,52]. Notably, RCS analysis revealed that the MeanSpO_2_ risk curves plateaued within the 88%-92% range, indicating that mean values fail to capture transient desaturation events and lack diagnostic sensitivity without complementary parameters [53]. TotalPower reflects global signal fluctuations without a specific mechanistic link to respiratory events [54]. These nonlinear parameter-risk relationships explain the suboptimal performance of linear models such as LR [10,23,44,55]. The steep threshold effects for ODI and HB suggest that even mild OSA can trigger substantial risk escalation, supporting the hypothesis of a critical threshold for hypoxic exposure [56,57].

### Multidimensional Synergy of Oximetric Indices

We further quantified interactions among multiple oximetric indices. While ODI tracks event frequency, it neglects desaturation depth and duration, failing to capture physiological nuances of OSA heterogeneity [10,57]. Our 2-parameter ODI-HB CatBoost model achieved an *F*_1_-score of 0.9472. By characterizing both temporal and intensity dimensions, this synergy explains why ODI, as the strongest predictor, requires HB integration to improve performance [57,58]. The superior performance of our primary model, ODI-HB-MinSpO_2_-ST90 (*F*_1_-score = 0.9516, AUC = 0.9879), stems from the inherent complementarity of these parameters. MinSpO_2_ captures severe hypoxic nadirs [16], while ST90 quantifies nocturnal hypoxemia duration [59]. Together, this 4-parameter ensemble facilitates comprehensive multidimensional phenotyping of OSA, encompassing the frequency, depth, duration, and cumulative burden of hypoxic events [57,60]. In terms of benchmarking, our model outperformed the multidimensional oximetry approach proposed by Kong et al [8] (AUC=0.939) and the least squares boosting model (AUC=0.889-0.924) developed by Gutiérrez-Tobal et al [23]. Moreover, integrated models incorporating demographics, questionnaires, and facial photography achieve AUCs ranging from 0.88 to 0.89 [1,61], whereas our oximetry-only approach achieved superior accuracy without auxiliary clinical data. This suggests multidimensional oximetry indices serve as effective PSG surrogates, encapsulating more direct pathophysiological information than traditional clinical markers [4].

### Benchmarking of Prediction Models and ML Algorithms

ML algorithms demonstrate variable performance in OSA screening [62]. LR yielded an AUC of approximately 0.77 [63], whereas SVM exhibited the highest accuracy exclusively for mild OSA [62]. RF reached 84.4% accuracy in predicting severe OSA but remained insensitive to complex feature interactions [62,64]. In contrast, gradient boosting frameworks excelled at handling heterogeneous interactions, class imbalances, and missing data [4,23]. By implementing ordered boosting, CatBoost effectively mitigates gradient bias, thereby enhancing model robustness and consistently outperforming established benchmarks such as XGBoost and LightGBM [4,65,66]. Our study represents the first application of CatBoost to multidimensional oximetry-based OSA screening, underscoring its capacity to resolve complex nonlinear relationships [24,25]. Notably, performance did not improve with 5 or more oximetry parameters. Indiscriminate feature addition introduces multicollinearity and overfitting without incremental gains [4]. By prioritizing core parameter selection over feature stacking, our model ensures both robustness and clinical feasibility, providing a basis for developing portable screening devices [7,23].

### Sex- and Age-Specific Performance Heterogeneity

Given the substantial phenotypic heterogeneity of OSA, sex-specific differences have been inadequately addressed in existing models, often leading to underdiagnosis among women [67-69]. To address this, we conducted stratified analyses by sex and age. In males and older subgroups, the ODI-HB-MinSpO_2_-ST90 model proved optimal, consistent with our overall findings. This result likely attributable to our predominantly male sample (median age 45 years). Conversely, the ODI-HB-MinSpO_2_-MeanSpO_2_ configuration demonstrated superior performance in females. Patiño et al [70] reported that despite lower AHI values, women exhibit mean SpO_2_ reductions comparable to those in men, while Poka-Mayap et al [71] confirmed significantly lower mean SpO_2_ levels in women with OSA, suggesting that the female OSA phenotype may be more closely linked to sustained hypoxemia. This may reflect heightened hypoxic sensitivity in women, where SpO_2_ fluctuations at subclinical AHI thresholds are sufficient to induce end-organ damage [56]. Notably, in older adults, the ODI-HB-MinSpO_2_-ST90 model achieved an exceptional AUC of 0.9950, surpassing that in younger participants. This enhanced accuracy likely stems from reduced hypoxic tolerance and pronounced SpO_2_ variability characteristic of aging. Indeed, older patients, particularly older women, consistently exhibit lower SpO_2_ levels independent of AHI burden [72].

### Model Interpretability and External Validation Robustness

Previous ML-based OSA diagnostic studies have focused predominantly on predictive performance, frequently neglecting to quantify parameter contributions, thereby limiting clinical trust and adoption of oximetry-based models [17,19,23]. We addressed this “black-box” limitation through SHAP values and feature importance analysis, which corroborated the central role of ODI and HB while revealing significant feature hierarchy shifts across subgroups. Notably, the importance of MeanSpO_2_ increased substantially in the female subgroup, reinforcing hypotheses regarding sex-specific physiological signatures in which sustained hypoxemia may characterize the female phenotype more prominently [56,69,71]. Furthermore, our CatBoost-based model maintained exceptional performance across independent external validation sets, with sex- and age-specific models demonstrating sustained stability, attributable to CatBoost’s ability to mitigate gradient bias [73]. Despite the relatively small non-OSA control group in the external validation cohort (n = 76), the high precision maintained across populations indicates strong potential for cross-cohort generalizability [7,23,74]. This robustness suggests that ML-integrated multidimensional oximetry can streamline OSA screening protocols and reduce reliance on resource-intensive PSG [75].

### Limitations

This study has several limitations. First, despite the large sample size, participants were recruited from a single center and comprised individuals referred for suspected OSA. This may have resulted in a higher OSA prevalence than in the general population, thereby increasing the pretest probability and potentially overestimating diagnostic performance. Second, the lack of longitudinal follow-up precludes assessment of the model’s temporal consistency or long-term predictive capacity. Third, our cohort lacked racial and ethnic diversity, and as skin pigmentation can introduce systematic biases in SpO_2_ measurements, this may limit generalizability to individuals with darker skin tones [76]. Finally, the single-center design and absence of prospective community-based validation restricts broader external validity.

### Conclusions

In conclusion, we developed and validated a robust CatBoost-based multidimensional oximetry model that enables accurate OSA screening. Although the ODI-HB-MinSpO_2_-ST90 combination demonstrated optimal performance in the general population, substituting ST90 with MeanSpO_2_ proved superior for female and younger subgroups. By integrating nocturnal hypoxemia indices spanning frequency, depth, and duration, our approach overcomes the limitations of single-parameter screening and offers multidimensional physiological assessment beyond conventional AHI-centric methods. These models can be readily integrated into portable monitoring devices or wearable technologies to facilitate early OSA diagnosis. Future research should prioritize multicenter prospective trials and multi-ethnic validation studies to establish standardized protocols for personalized OSA risk stratification.

## Abbreviations

AHI: apnea-hypopnea index
AUC: area under the receiver operating characteristic curve
CatBoost: categorical boosting
HB: hypoxic burden
HSAT: home sleep apnea test
LightGBM: light gradient boosting machine
LR: logistic regression
ML: machine learning
ODI: oxygen desaturation index
OSA: obstructive sleep apnea
PPV: positive predictive value
PSG: polysomnography
RCS: restricted cubic spline
RF: random forest
SHAP: Shapley additive explanations
SMOTE: synthetic minority over‑sampling technique
SVM: support vector machine
XGBoost: extreme gradient boosting

## Data Availability

The datasets generated or analyzed during this study are not publicly available due to privacy concerns, ethical restrictions, and protected health information regulations but are available from the corresponding author on reasonable request. The underlying code for this study (and training/validation datasets) is not publicly available but may be made available to qualified researchers on reasonable request from the corresponding author.
